# Robotic enucleation for pediatric insulinoma with MEN1 syndrome: a case report and literature review

**DOI:** 10.1186/s12893-018-0376-5

**Published:** 2018-06-19

**Authors:** Mei Liang, Jialin Jiang, Hongmei Dai, Xiafei Hong, Xianlin Han, Lin Cong, Anli Tong, Fang Li, Yaping Luo, Weinan Liu, Liangrui Zhou, Wenyu Di, Wenming Wu, Yupei Zhao

**Affiliations:** 1 Department of Surgery, Peking Union Medical College Hospital, Chinese Academy of Medical Science & Peking Union Medical College, Beijing, China; 2 Department of General Surgery, Peking Union Medical College Hospital, Chinese Academy of Medical Science & Peking Union Medical College, Beijing, China; 3 Department of Endocrinology, Peking Union Medical College Hospital, Chinese Academy of Medical Science & Peking Union Medical College, Beijing, China; 4 Department of Neurology, Peking Union Medical College Hospital, Chinese Academy of Medical Science & Peking Union Medical College, Beijing, China; 5 Department of Pathology, Peking Union Medical College Hospital, Chinese Academy of Medical Science & Peking Union Medical College, Beijing, China; 6 Department of Pathology, The First Affiliated Hospital of Xinxiang Medical University, Weihui, China

**Keywords:** Pediatric insulinoma, Robotic enucleation, MEN1 syndrome, Surgery

## Abstract

**Background:**

A patient with a rare pediatric insulinoma and MEN1 syndrome was treated by robotic enucleation surgery.

**Case presentation:**

We present a case of a 9-year-old girl presenting with repeated loss of consciousness, concomitant with a pale face, palpitations, and convulsions, which had persisted for 2 years and had been aggravated during the previous 2 months. She was previously misdiagnosed with epilepsy in another hospital. We further examined her while she was hospitalized. By combining her medical history and imaging examination and lab test results, a diagnosis of insulinoma was confirmed. Sanger-directed sequencing on a peripheral blood sample revealed an MEN1 gene mutation, indicating pediatric insulinoma with MEN1 syndrome. The patient underwent minimally invasive insulinoma enucleation surgery under the Da Vinci robot-assisted system with intraoperative ultrasound (IOUS) connected. The surgery was successfully completed within 65 min, and the girl recovered well postoperatively and no longer experienced symptoms of hypoglycemia.

**Conclusion:**

This is the first report of a case of pediatric insulinoma treated using robotic enucleation. This experience demonstrates the feasibility and safety of combining robotic surgery with the enucleation procedure as an excellent strategy for pediatric insulinoma.

## Background

Insulinomas are insulin-secreting pancreatic neuroendocrine tumors (pNETs) that affect an estimated 1 in 250,000 people per year. The median age of patients at presentation is approximately 47 years, and insulinomas seldom occur in pediatric patients [1]. Due to excess insulin secretion, patients with insulinomas have recurrent hypoglycemia. Such patients usually present with Whipple’s triad, which consists of hypoglycemia, neuroglycopenic symptoms, and symptom relief with glucose administration. Nearly 10% of insulinomas occur in the context of MEN1, an autosomal dominant disorder. More than 1300 mutations in MEN1 have been reported, and most of these mutations likely disrupt the interactions of the menin protein with other proteins, thus altering critical events in cell cycle regulation and proliferation [2]. Additionally, MEN1 mutations are more common in insulinomas in children than in adults [3]. Diagnoses of insulinoma can be challenging because of the presence of nonspecific symptoms. Surgical resection is the best choice for most patients. In this paper, we describe a patient with pediatric insulinoma and MEN1 syndrome. Traditionally, open surgery has been the main surgical choice for such a pediatric patient. Recently, minimally invasive surgery has been adopted to treat insulinoma. Ming-Gen et al. recently used robotic spleen-preserving distal pancreatectomy to treat pediatric insulinoma [4]. Our surgical center was the first to report a study on robotic enucleation for small pancreatic neuroendocrine tumors in adults [5]. Here, we present robotic enucleation for a pediatric insulinoma patient with MEN1 syndrome. To the best of our knowledge, this is the first reported robotic enucleation surgery for pediatric insulinoma.

## Case presentation

**Fig. 1:**
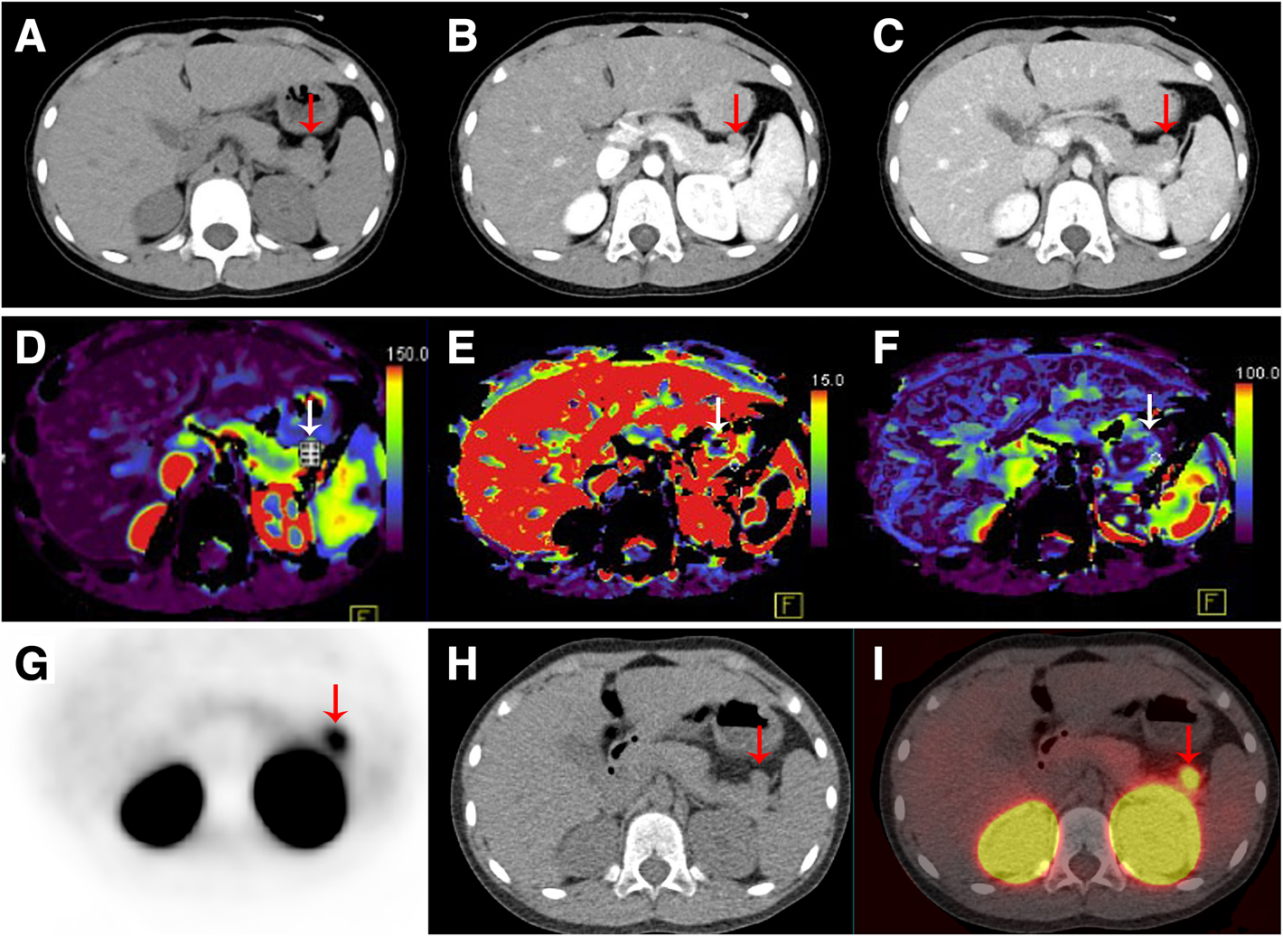
CT and ^68^Ga-exendin-4 PET-CT scan of the pancreas. The tumor is marked with asterisks. **a** Non-enhanced phase scan. **b** Arterial phase. **c** Portal phase. **d**-**f** Pancreatic perfusion imaging. **g**-**i** ^68^Ga-exendin-4 PET-CT scan of the pancreas

A 9-year-old girl was admitted due to repeated loss of consciousness, concomitant with a pale face, palpitations, and convulsions, which had persisted for 2 years and had been aggravated during the previous 2 months. These symptoms occurred automatically. The patient denied experiencing any sweating, nausea, vomiting, trembling, or an obvious sense of hunger before meals. The patient was previously misdiagnosed with epilepsy in another hospital, but no abnormal findings were detected on a 24-h electroencephalogram at our hospital. Her abdominal perfusion CT showed a highly perfused nodule within the pancreatic tail; A magnetic resonance scan confirmed the location of this nodule and indicated that its size was 11.6 × 13.2 mm (Fig. 1a-f). Additionally, ^68^Ga-exendin 4 PET-CT showed a region in the pancreatic tail with abnormally high metabolism and overexpression of the glucagon-like peptide-1 receptor (Fig. 1g-i). Lab testing showed a low fasting blood glucose (BG) of 2.2 mmol/L (reference range: 3.9–6.1 mmol/L), a high proinsulin level of 4455.9 pg/mL (reference range: 30–180 ng/mL), a normal C-peptide level of 2.56 ng/mL (reference range: 0.8–4.2 ng/mL), a serum insulin level of 15.35 μIU/mL (reference range: 5.2–17.2 μIU/mL), and a gastrin level of 92.6 pg/mL (reference range: < 100 pg/mL). These results confirmed a diagnosis of insulinoma. Imaging examination showed no abnormalities indicative of parathyroid adenoma or malignancy in the pituitary or adrenal glands. Lab testing showed normal levels of parathyroid hormone (PTH), blood calcium, phosphate, follicle-stimulating hormone (FSH), growth hormone (GH), prolactin (PRL), adrenocorticotropic hormone (ACTH), 24-h urinary free cortisol (24 hUFC), and serum cortisol. Her luteinizing hormone (LH) level was 0.24 IU/L (reference range: 2.12–10 IU/L during the follicular phase), which was considered related to her age.

Preoperative preparation: To avoid recurrent symptoms and to maintain her fasting BG at a tolerably low level, the patient was given regular snacks before bedtime. BG can be controlled at a level between 50 and 60 mg/dL preoperatively.

**Fig. 2:**
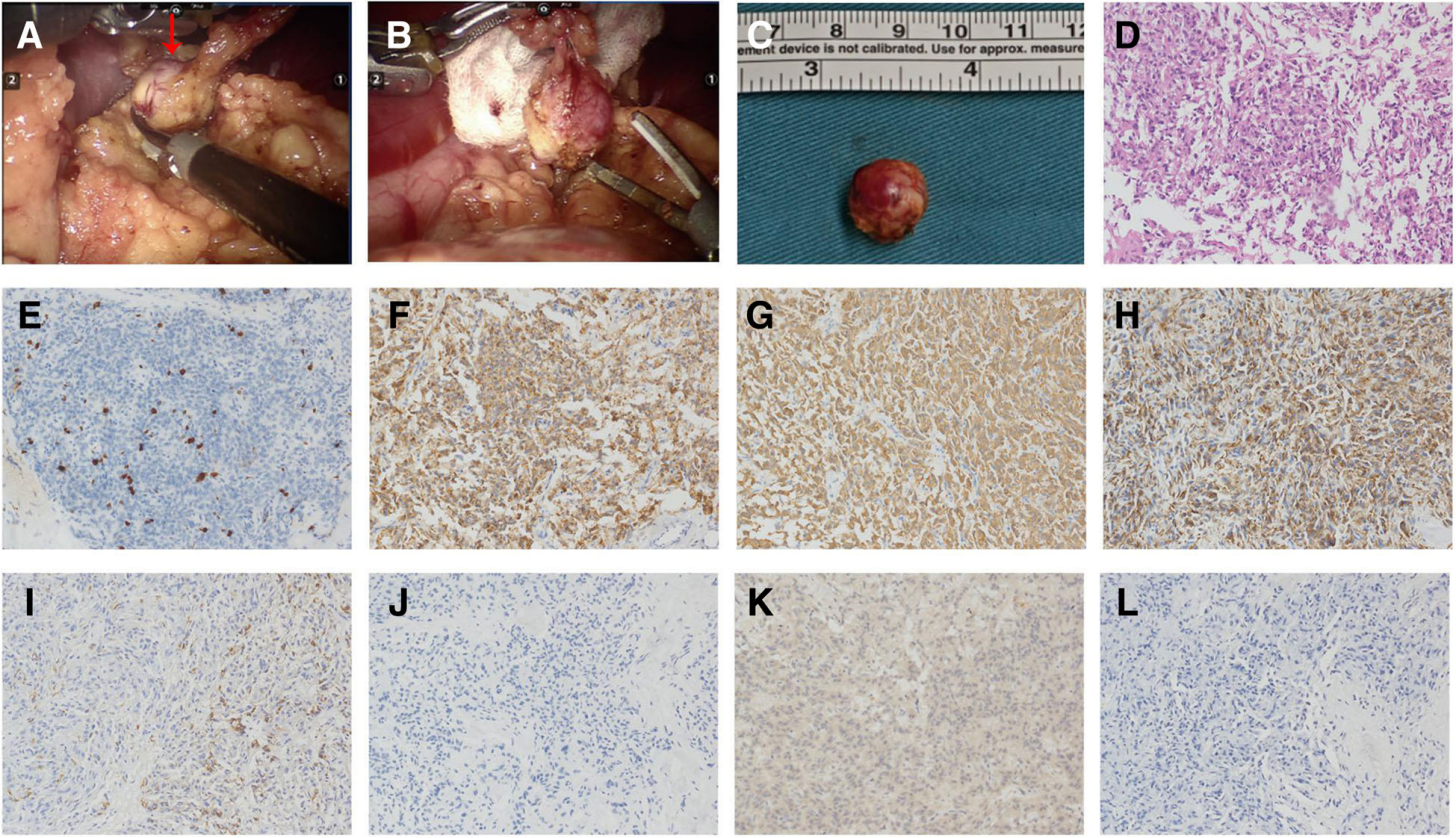
Surgical and pathology images of the tumor. **a**-**b**: Surgical photographs of pancreatic tumor enucleation. **c**: Gross view of the tumor. **d**-**e**: HE and Ki-67 staining, which showed that the pancreatic neuroendocrine tumor was of Grade 2 with a Ki-67 index of 4%. **f**-**h**: CgA, Syn, and AE1/AE3 staining were positive, which confirmed that the tumor was derived from neuroendocrine cells. i: Insulin staining was partially positive. **j-l**: Glucagon, gastrin and somatostatin staining were negetive

Surgical procedure: The patient underwent minimally invasive insulinoma enucleation surgery under the Da Vinci robot-assisted system with intraoperative ultrasound (IOUS) connected. The patient was put in a head-low, feet-high and left-lateral position. The robotic system was positioned at the head of the patient, while the assistant surgeon stood between the patient’s legs. Abdominal exploration via laparoscopy was conducted, and no obvious abnormalities were found. The robotic lens and operating arms were docked. The gastrocolic ligament was dissected with an ultrasonically activated scalpel. The head of the pancreas was exposed by grasping the colon downward and lifting the stomach. Towards the tail of the pancreas, we separated and exposed the spleen. The surgeon then controlled the ultrasound probe, exploring the tumor from the pancreatic tail to the head and the uncinated process with the assistance of a prograsp clamp. A quasi-circular, hypoechoic lesion was found at the end of the pancreas with a diameter of approximately 10 mm and a clear boundary. We marked the normal pancreatic tissue around the lesion with an electrotome, and while dividing the pancreas sequentially, suction was used continually to visualize the tumor capsule. Precise positioning was achieved using IOUS, and the tumor was completely resected along the capsule (Fig. 2a-c). A peritoneal drainage tube was placed. The surgery went well, lasting 65 min (skin to skin), and the volume of intraoperative bleeding was 5 mL. Intraoperative BG is documented in Table 1.

**Table 1:** Intraoperative blood glucose monitoring

Time (minutes)	Blood Glucose (mg/dL)
Prior to tumor removal	59.4
Immediately following removal of the tumor	68
15 min after tumor resection	82.8
30 min after tumor resection	90
40 min after tumor resection	75.6
60 min after tumor resection	90

After surgery, the patient was given liquid diet on POD2. The drain was clean and was removed on POD4, and the patient gradually resumed her normal diet. She was discharged to home on POD6. During the following 1.5 years, the patient had no recurrence of the disease. No postoperative complication occurred, such as pancreatic fistula or pancreatic function deficiency.

Pathological examination showed that the tumor was a pancreatic neuroendocrine tumor (Grade 2 with a Ki-67 index of 4%) (Fig. 2d-e). This tumor was positive for CgA, Syn, and AE1/AE3 (Fig. 2f-h). Insulin staining was partially positive (Fig. 2i), while gastrin, glucagon, and somatostatin staining were negative (Fig. 2j-l).

**Fig. 3:**
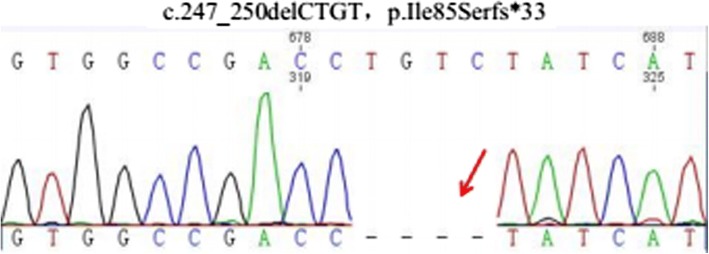
Sequence analysis of the MEN1 gene revealed a homozygous frameshift for c.247_250delCTGT(p.Ile85Serfs∗33). This point mutation was detected both in the blood sample and frozen tissue of the patient

Sanger-directed sequencing for the MEN1 gene mutation was performed on a peripheral blood sample, revealing a homozygous pathogenic mutation of c247_250delCTGT (p.Ile85Serfs*33) (Fig. 3). This point mutation was also detected in the frozen tissue of the patient.

## Discussion and conclusion

Insulinoma is the most common type of pNET, and such tumors seldom occur in pediatric patients [6]. Recently, we searched all of the literature regarding pediatric insulinoma from the past 10 years and summarized the characteristics of 65 cases in 16 studies (Table 2) [4, 7–21].

**Table 2:** Published Cases (Surgery*: Details of surgery not mentioned)

Publication year	Author	Country	Number	Age, years	Diagnosis	Main location method	Tumor size (cm)	Lesion location	Treatment
2017	Ming-Gen Hu [4]	China	1	9	Solitary	MRI	2	Tail	Robotic distal pancreatectomy
2017	Esposito C [7].	Italy/UK/Netherlands/France	4	0.16–4	Solitary	CT, ^18^F-DOPA PET/CT	N/A	Body/tail	Laparoscopic
2016	Miron I [8]	Romania	1	11	Solitary	MRI	1	Tail	Open surgery
2016	Bhatti TR [9]	America	12	4–16	Multiple: 2; MEN1: 5	N/A*	0.7–2	Head/neck/midbody/tail/ splenic hilum	Diazoxide, surgery*
2015	Nasher O [10]	UK	3	0.09–10	Solitary: 2; Multiple: 1	N/A	N/A	Head/tail	Laparoscopic to open surgery: 1; open surgery: 2
2015	Smith A [11]	USA	1	14	Solitary	ASVS	2.1	Neck	Diazoxide, surgery*
2014	Gozzi Graf T [12]	Switzerland	2	11.3–13.6	Solitary: 1	MRI, ASVS*	1.2–2.5	Head	Laparoscopic: 1; open surgery: 1
2014	Padidela R [13]	UK	9	2–14.5	Multiple: 1; MEN1: 2	MRI/^18^F-DOPA PET/CT	0.8–2	Head/uncinate process/tail	Diazoxide, surgery*
2014	Kundel A [14]	Ireland	6	12–18	N/A	N/A	N/A	Tail	Surgery*
2013	Horváth E [15]	Romania	1	16	Solitary	CT	1.6 cm	Tail	Surgery*
2013	Peranteu WH [16]	USA	8	Mean: 11	Solitary: 7; Multiple: 1; MEN1: 1	U/S, CT, MRI, endoscopic U/S, ASVS, THPVS*, ^18^F-DOPA PET/CT	0.3–1.8	Head/neck/body/tail	Open surgery
2012	Sakusai A [17]	Japan	13	< 20	Solitary/multiple, all MEN1	N/A	0.15–9.4	Head/body/tail	Laparoscopic/open surgery
2012	Ide S [18]	Japan	1	13	Solitary	Enhanced CT	1.9	Head	Open surgery
2010	Janem W [19]	Jordan	1	12	Malignant	CT	N/A	Widely metastatic	Octreotide, diazoxide
2008	Shah SR [20]	India	1	13	Multiple	N/A	2	Tail	Surgery*
2007	Bonfig W [21]	Germany	1	12.5	Solitary	Endosongraphy	1.5	Tail	Laparoscopic

Among the cases in the past decade that we reviewed, distal pancreatectomy and pancreaticoduodenectomy were the most common therapeutic procedures. Only 4 enucleations were performed, including 3 open and 1 laparoscopic. Notably, one patient underwent robotic distal pancreatectomy in 2017, which was the first application of a robotic surgery system in pediatric insulinoma treatment.

Imaging examination is an essential step for preoperative confirmation and localization of an insulinoma. In our case, we used two advanced methods for localization which have improved the accuracy of diagnosis significantly: ^68^Ga-DOTANOC PET/CT and laparoscopic ultrasound (LUS). An examination of pNET diagnosis and staging data from 141 patients indicated that the overall sensitivity, specificity, and accuracy of ^68^Ga-DOTANOC PET/CT for diagnosing patients with pancreatic neuroendocrine tumors were 85.7, 79.1, and 84.8%, respectively [22]. Meanwhile, in a study by Li et al., intraoperative LUS alone detected all insulinoma tumors with 100% sensitivity and 100% specificity [23].

In the past, pancreaticoduodenectomy and pancreatectomy have generally been the standard operative procedures for benign and borderline pancreatic tumors, but they seem excessive because of the increased risk of postoperative endocrine and exocrine insufficiency owing to wide resection of the pancreatic parenchyma. Patients undergoing partial resection had a higher rate of new postoperative diabetes and more use of pancreatic enzymes postoperatively than those who receiving pancreatic enucleation [24] .

Therefore, enucleation appears to be a better option as an effective therapeutic procedure for such tumors, allowing preservation of long-term pancreatic function while achieving favorable oncological outcomes [25]. Previous reports have shown that enucleation allows a more limited excision extent, thus offering several advantages in terms of function conservation, blood loss and surgical time [26], all of which are especially important for children whose normal parenchyma is still developing. Additionally, many studies have confirmed the low recurrence rate after enucleation of benign or low-grade malignant tumors. No significant difference is evident in prognosis of patients with insulinoma between groups of enucleation and partial pancreatic resection [27].

Specific indications for pancreatic tumor enucleation are as follows: benign, isolated lesions with a distance between the tumor and the main pancreatic duct ≥3 mm (no focal stricture or dilation), insulinomas, gastrinomas < 2 cm, and nonfunctional pancreatic neuroendocrine tumors (NF-pNETs) < 1–2 cm with a low Ki67 mitotic index [28]. Insulinomas < 2 cm are ideal candidates for enucleation, especially considering their 80% probability of being benign [28].

The Da Vinci Surgical System enables robot-assisted surgery, which provides surgeons with a three-dimensional view, high articulation ability, scaling functions, filtering functions to avoid shaking, and visual magnification. When it comes to pancreatic surgery, several studies have reported the advantages of robotic approach over conventional laparoscopic means, such as less rate of conversion to laparotomy, less blood loss and shorter postoperative hospital stay [29, 30]. However, enucleation of pNETs via robotic surgery has been described only a few times worldwide. Recently, our surgical center reported that robotic surgery for enucleation of pNETs smaller than 2 cm did not increase postoperative pancreatic fistula (POPF) or major complication rates and reduced the duration of surgery and estimated blood loss when compared with open surgery [5]. ,Robotic enucleation provides the dual benefits of minimal invasiveness and good pancreatic parenchymal conservation. Notably, for children, considering their poor tolerability for operative trauma and anesthesia, demands for a faster and less traumatic surgical procedure are increasing. Therefore, robotic enucleation is considered an ideal strategy for pediatric insulinomas meeting the operative indications.

This is the first case of pediatric insulinoma treated via robotic enucleation in the world. The operation was completed in 65 min (skin to skin) with only 5 mL of blood loss. No POPF occurred after surgery. The patient recovered well and was discharged home on POD 6. During the following 1.5 years, she had no recurrence of the disease nor experienced her previous symptoms of hypoglycemia. No postoperative complication occurred, such as pancreatic fistula or pancreatic function deficiency. This experience has demonstrated the feasibility and safety of the robotic enucleation procedure, with an excellent curative effect for pediatric insulinoma.

## Abbreviations

ASVS: Selective arterial calcium stimulation and hepatic venous sampling of insulin
MEN1: Multiple endocrine neoplasia type 1
N/A: Data not available
PET-CT: Positron Emission Tomography-Computed Tomography
POD: Postoperative day
POPF: Increase postoperative pancreatic fistula
THPVS: Transhepatic portal venous sampling
